# Comparing the Current Training Running Demands of Elite Camogie Players to Competitive Match-Play

**DOI:** 10.3390/sports10080113

**Published:** 2022-07-28

**Authors:** Philip Connors, Declan Browne, Des Earls, Paula Fitzpatrick, Paula Rankin

**Affiliations:** HealthCORE, Department of Health and Sport Sciences, South East Technological University, R93 V960 Carlow, Ireland; declan.browne@setu.ie (D.B.); des.earls@setu.ie (D.E.); paula.fitzpatrick@setu.ie (P.F.); paula.rankin@setu.ie (P.R.)

**Keywords:** team sports performance, training load, intermittent exercise, Gaelic games

## Abstract

Training to meet match-play demands is a primary objective in an athlete’s preparation for their games. Despite camogie match-play running demands being available, how current training practices compare, specifically individual training components, remains unknown. This study aimed to investigate and compare current elite camogie training to match-play demands. Thirty-three (n = 33) elite camogie players wore 10 Hz Playertek GPS units during twenty-five training sessions and ten competitive matches. Training sessions were analysed using ball-in-play time and split into warm-ups, drills, small-sided games, running, and training matches. Metrics were converted into relative terms (per minute), to allow comparisons. Players cover significantly greater (*p* < 0.05) total distance (non-parametric standardised effect sizes (r = 0.45)), peak speed (r = 0.45), high-speed running (r = 0.13), sprint distance (r = 0.20), and total decelerations (r = 0.12–0.22) during match-play than training. Relatively, players cover significantly greater distance during running, small-sided games, and training matches compared to match-play (r = 0.21–0.29). Compared with match-play, running results in significantly greater high-speed running and sprint distance (r = 0.18–0.41), with greater accelerations (3–4 m∙s^−2^) during warm-ups, running, and small-sided games (r = 0.14–0.28). Current total training demands seem to fall behind match-play. However, relatively, training matches and small-sided games match or surpass competitive match-play demands. These findings may be utilised in preparing camogie teams for competition.

## 1. Introduction

Camogie is a national female Gaelic sport, indigenous to Ireland. It is played on a grass pitch 145 m in length, and 90 m in width, with teams comprised of fifteen players each: one goalkeeper, six defenders, two midfielders, and six forwards. Games are sixty minutes in duration, consisting of two halves of thirty minutes, interspersed by a halftime interval of ten minutes. Players use a stick (hurl), made from ash, to hit a small leather ball (sliotar), with the aim of the game to outscore the opposition by scoring points (above the crossbar), or goals (below the crossbar), which are worth three points. The primary competitions each year are the National League and All-Ireland Championship, traditionally taking place from January to April, and May to September, respectively.

To date, camogie research has reported on players’ injury rates [1,2], strength and conditioning recommendations [3], match-play demands [4,5,6], and the physical characteristics of the players [7]. Gaining a greater understanding of match-play demands and players’ physical capabilities allow more informed training practices to occur. This ensures players are adequately prepared, limiting potential injuries, which may occur due to overtraining [8], as well as optimizing performance by ensuring a super compensatory effect through a correct training dose–response. 

Previous camogie match-play demand research reports that elite camogie players cover 5881 ± 906 m (98 ± 15 m∙min^−1^), with 546 ± 259 m and 183 ± 130 m covered at high-speed and sprint speeds, respectively, during a 60 minute competitive match [5]. Furthermore, elite camogie players are reported to achieve mean peak speeds of 6.86 ± 0.35 m∙s^−1^ during match-play [4], with positional differences noted across metrics. However, despite these data being available, it remains unknown whether current training methods and specific individual training components are adequately preparing players for match-play. Without this knowledge, it is possible players may be left under-prepared, or even over-trained, due to the unknown quantities of current practices. 

Training is defined as engaging in an activity to improve performance and fitness [9]. Different methodologies are frequently used in field sport athlete training. The literature on coaching details two predominant methods that coaches utilise when designing training sessions. These are the drill-to-game approach, traditionally used in coaching practice [10], and the games-based approach (GBA). The GBA is associated with game-play activities that require players to perform game-specific skills under pressure, thus, requiring more frequent decision-making [11,12]. Gaelic games research seems to suggest coaches employ a combination of both methods [13], with the time of season seeming to dictate the preferred approach. Thornton et al. [14] report greater distances at higher intensities being covered during matches than drills, with no differences evident between matches and conditioning in a cohort of female Australian Rules players. Furthermore, Tee et al. [15] report that high-intensity interval training elicits similar running demands to match-play in male rugby players, with game-based training failing to meet match-play intensity across positions, with moderate to large effects. 

Small-sided games are an effective method of improving physical fitness performance in male hurlers compared to continuous aerobic training, whilst also being more time-efficient [16]. Furthermore, a periodised small-sided games intervention seems to improve both physical and physiological fitness parameters of performance in another cohort of hurlers [17]. Therefore, game-based methods may be a worthwhile training methodology to improve physical fitness in Gaelic games. Recent research suggests that small-sided games are an effective method of also improving technical skills [18]. Drill-based sessions with opposition present also result in players covering greater distances, reporting a higher rate of perceived exertion, and having a higher relative intensity than closed drills [19]. 

Despite the benefits associated with different training methods, their ability to match competitive camogie match-play demands currently remains unknown. This investigation aims to redress this, and quantify the current training running demands of elite camogie players. The hypothesis is that small-sided games and training matches may elicit similar running demands to that of match-play, with drills-based training resulting in lower training loads. 

## 2. Materials and Methods

### 2.1. Experimental Approach to the Problem

To investigate and compare the current elite inter-county camogie physical training demands to competitive match-play, a squad of inter-county camogie players competing in the Senior All-Ireland Championship and Division 1 of the National League was recruited. The study was an observational longitudinal research design. 

### 2.2. Participants 

Thirty-three (n = 33) elite female camogie players (age: 25 ± 3 years; height: 186.2 ± 6.5 cm; body mass: 64.4 ± 3.9 kg) from a senior inter-county camogie squad volunteered to participate in this investigation. Players who participated in this investigation completed 3–4 collective training sessions per week, with sessions 1–1.5 hours in duration. All players had at least one year of training experience at inter-county level. Although players were not broken into their positional groups, players from each positional group of defenders, mid-fielders, and forwards were included. Ethical approval was sought and granted by the Institutes’ Research Ethics Committee (Protocol No. 253). Health screening questionnaires and institutionally approved informed consent forms were completed by all players, where the benefits and risks associated with the investigation were disclosed. Players were informed they were free to withdraw at any time. All data collected conformed to the Declaration of Helsinki recommendations. 

### 2.3. Training and Matches 

Training sessions and matches from both the League and Championship competitions were monitored across a season. A total of 25 training sessions (11 during the League and 14 during Championship) and 10 matches (5 League and 5 Championship) were monitored, and subsequently analysed. Total training was analysed using ball-in-play time, which comprised of warm-ups, drills, small-sided games, running, and training matches. Warm-ups, drills, small-sided games, running, and training matches were also individually analysed, with transitions also marked, all of which are defined in Table 1. These are in line with the breakdown of training components used by Thornton et al. [14] in their Australian Rules research. However, further to the breakdown used by Thornton et al. [14], this investigation also added training matches, to compare their demands to competitive match-play. A total of 3942 individual datasets were collected (warm-up (n = 377), drills (n = 1756), small-sided games (n = 1000), running (n = 365), training matches (n = 349), and competitive matches (n = 95)). Transitional periods were excluded from analysis, given their similarity to the half-time period of match-play. 

Running performance was monitored using commercially available 10 Hz Playertek GPS units, integrated with a 400 Hz triaxial accelerometer, and a 10 Hz triaxial magnetometer (*Playertek by Catapult, Australia*). The validity and reliability of 10 Hz GPS units on distances and velocities across linear and team-sport circuits were frequently reported in previous investigations [20]. The 10 Hz units also exhibited good to moderate inter-unit reliability for acceleration and deceleration metrics [21]. The unit (dimensions: 85 mm × 40 mm × 20 mm) was placed in a protective pouch between the players’ shoulder blades, in the upper thoracic region. GPS units were switched on 15 minutes prior to the warm-up of each match or training session, to ensure a satellite connection was established. 

Total distance (TD) (km), relative distance (RD) (m∙min^−1^), peak speed (m∙s^−1^), high-speed running distance (HSR distance) (m), sprint distance (m), total accelerations at 3–4 m∙s^−2^ and >4 m∙s^−2^, and total decelerations at 3–4 m∙s^−2^ and >4 m∙s^−2^ were analysed to quantify running performance during training and matches [22]. HSR and sprint distance thresholds were set at 4.4 m∙s^−1^–5.5 m∙s^−1,^ and >5.5 m∙s^−1^, respectively, in line with previous research [23]. Data analysis was conducted by retrospectively downloading the data from the Playertek software to a Microsoft Excel spreadsheet (Excel, Redmond, WA, USA). Only players who completed a full training session or match were included in the data analysis. 

Training session relative metrics were calculated using the mean total of the training component, divided by the mean time spent on that component. 

### 2.4. Statistical Analysis

All analysis was conducted using IBM SPSS Software 27. All data were assessed for normality using Shapiro–Wilks test for normality. Data violated normal distribution, therefore, comparisons between total match-play and total training were assessed using Mann–Whitney U test. 

Total match-play and training (ball-in-play) are reported as median (interquartile range). Relative running demands were calculated by dividing the total value by the time spent on each training component. Relative comparisons between training components and that of competitive match-play were assessed using Kruskal–Wallis H test, with pairwise comparisons and Bonferroni adjustments. Relative descriptive statistics are reported as median (interquartile range). Significance was set at the accepted alpha level of *p* < 0.05. To calculate the magnitude of effect, non-parametric standardised effect sizes (r) were calculated by dividing the standardised test statistic by the square root of the sample size [24]. Effect sizes were interpreted using Cohen’s d estimate of effect size interpretations: trivial (r < 0.20), small (r = 0.20–0.49), moderate (r = 0.50–0.79), large (r = 0.80–1.00) [25]. Furthermore, total training and training components were expressed as a percentage of match-play data.

## 3. Results

Mean training session time (hh:mm:ss) is 01:10:44 ± 00:19:37, of which ball-in-play time is 00:43:02 ± 00:15:01. The median ball-in-play total and relative distance covered during training is 4.25 km (2.75–5.50 km), and 96 m∙min^−1^ (88–104 m∙min^−1^), respectively. The following time is spent on each training component as a percentage of the total session; warm-up: 12.2%, running: 5.6%, drills: 14.1%, small-sided games: 12.2%, training matches: 16.8%, transitions: 39.1%. Running performance metrics for training and match-play data are reported as median (interquartile range) in Table 2, with relative running performance for each training component reported as median (interquartile range) in Table 3.

Significant differences between total match-play and total training (ball-in-play) for all metrics are observed. Players cover significantly greater (*p* < 0.05) total distance (r = 0.45), peak speed (r = 0.45), high-speed running (r = 0.13), sprint distance (r = 0.20), and total decelerations across both zones (r = 0.12–0.22) during match-play than training. Relative distance and total accelerations in both zones are significantly greater during training than in match-play, however (*p* < 0.05) (r = 0.10–0.19).

The Kruskal–Wallis H test reveals significant differences that exist between training components and match-play (*p* < 0.05). Players cover significantly greater relative distance during small-sided games, running, and training matches compared to competitive match-play (r = 0.21–0.29, 122–127%). Furthermore, players cover significantly greater high-speed running and sprint distances during running drills compared to that of competition (r = 0.18–0.41, 294–371%); however, they cover less sprint distance during small-sided games (r = 0.16, 31%). Elite camogie players’ total accelerations at 3–4 m∙s^−2^ are significantly greater during warm-up, drills, small-sided games, and running than during match-play (r = 0.14–0.28, 129–236%), while running also has significantly greater total accelerations at greater than 4 m∙s^−2^ (r = 0.24, 409%). Players have significantly greater decelerations at 3–4 m∙s^−2^ during drills and small-sided games compared to match-play demands (r = 0.08–0.13, 136–154%), with warm-up, drills, and running having a significantly lower number of decelerations at greater than 4 m∙s^−2^ than match-play (r = 0.11–0.24, 0–55%). 

## 4. Discussion

This investigation is the first to report on the current running demands of elite camogie training with respect to total training and individual training components. Despite previous research on the competitive match-play demands [4,5,6], how current training practices compare to that of the match-play demands remain unknown. The analysis reveals total training running demands, and specific training components, to differ from match-play across metrics. 

Significantly greater TD, HSR distance, sprint distance, and total decelerations are noted during match-play compared to training. One potential reason for this may be due to the inclusion of training sessions that took place prior to match days, where it would be expected that training loads are tapered. However, Doyle et al. [26] noted the opposite to occur in elite international female footballers, with matchday minus two having very high volume and intensity in comparison to other training days. Therefore, it would appear current total training may not adequately prepare players for the demands of competitive match-play. However, the time of season may also play a role in when the training load matches the match-play demands. Total training loads similar to the match-play demands during the peak season may not be possible, given the risk of over-training, with it perhaps being more suitable during pre-season. Future research may look to investigate this further. Players completed significantly less high-speed running during training than match-play, a trend also reported previously in a cohort of professional soccer players [27]. However, players did cover a greater number of accelerations, as well as having significantly higher relative distance during training than match-play. 

When assessing individual training components, and comparing them to relative match-play demands, training matches (15 v 15 on a full pitch with standard regulations) equalled or surpassed competitive match-play running demands across all metrics. Similarly, small-sided games result in significantly greater relative distance and high-speed running, as well as accelerations and decelerations at 3–4 m∙s^−2^, compared with match-play. Relatively, both training matches and small-sided games compare favourably to match-play in this cohort of camogie players, which supports the original hypothesis of this investigation. Therefore, both may be seen as appropriate training methods to prepare players for the match-play running demands of elite competition. 

Small-sided games do not match the sprint distance associated with match-play though. This is likely due to the playing area not being large enough to allow players to reach higher velocities. It is possible to manipulate running demands during small-sided games by altering certain variables such as player numbers or pitch sizes [28]. Previous small-sided game research in a cohort of hurlers reveals larger pitch sizes result in players completing greater relative distance, high-speed, and very high-speed running, as well as total number of accelerations and accelerations distances, compared to small- and medium-size pitches [29]. Therefore, the implementation of larger games may result in players completing greater sprint distances more closely aligned with that of match-play. 

This investigation notes that the median peak speed during competition is 7.22 m∙s^−1^ (6.94–7.62 m∙s^−1^), with the median peak speed during ball-in-play training significantly lower than match-play, despite reaching 92% of match-play peak speeds. This potential lack of exposure to match-play peak speeds during training may result in the under-preparation of players for competitive peak speed demands. Given the role that high-speed running and sprinting play in goal-scoring situations, and match results in other sports [30], attempts to reach the match-play peak speeds in training sessions should be a priority.

Running, as defined in Table 1, results in significantly greater running demands across all metrics, except for accelerations at 3–4 m∙s^−2^. Thornton et al. [14] report similar results when comparing ‘conditioning’ against Australian Rules match-play demands. As a result, this mode of training may appear an effective method in preparing players for the match-play running demands. However, the absence of technical and tactical skills, decision-making, and, in some instances, game-specific movements when running is utilised, may result in players being under-prepared for critical elements of match-play, which this investigation did not seek to report on. 

Perhaps expected, warm-ups, given the sub-maximal elements associated with them, in comparison to match-play data, result in less relative distance, high-speed running, and sprint distance. This is in line with reports from Thornton et al. [14]. However, a point of interest may be the significantly greater number of accelerations at 3–4 m∙s^−2^ and decelerations performed at greater than 4 m∙s^−2^ during warm-ups compared to match-play. Acceleration plays an important role in field sport games [21], therefore, it is important players’ warm-ups prepare them accordingly.

When the transitioning phase of training is excluded, 47.70% of training time is spent on small-sided games and training matches. Given the results of this investigation, which seem to indicate the ability of both methods to surpass the relative match-play demands, this would seem to be an appropriate method utilised by the coaches. Perhaps given the lack of decision-making and technical skills required, running is not given the same priority by coaches, accounting for just 9.21% in comparison. 

Despite the benefits of utilising more game-based scenarios, drill-based training does equal the relative distance covered during match-play, as well as players covering significantly greater accelerations and decelerations at 3–4 m∙s^−2^. Therefore, coaches may wish to utilise this method in elite camogie training. Karahan et al. [31] report that skills-based training at maximal intensity may be more effective than small-sided games in improving performance in a cohort of young soccer players. The results of this investigation seem to conclude that small-sided games and training matches are more appropriate methods in preparing camogie players for inter-county camogie match-play. This agrees with previous training research, with such methods exposing the athlete to high-intensity work, while simultaneously developing the players’ skills [14]. This investigation was conducted with a single camogie squad, perhaps a limitation of the study given similar training methods and team tactics may have been utilised across the season. The absence of another measure of player monitoring, such as rate of perceived exertion or heart rate monitoring, may also be another limitation. Future research may be conducted with consideration for these limitations, while also investigating further the relationship between time of season and training load. Furthermore, researchers may also seek to report on the technical performance indicators of the sport during training and match-play.

## 5. Conclusions

This research provides insight into the current elite training practices. Differences between total match-play and total training, as well as between relative match-play and training components, are evident. 

Training matches equal or surpass relative match-play demands across metrics. The results reveal players do not reach match-play sprint distances through small-sided games, however. Increasing the playing area or reducing player numbers may aid in rectifying this, as manipulation of these variables leads to an increase in running. A combination of training matches and small-sided games appears to prepare players appropriately for the match-play demands. 

Although drills may seem to under-prepare players in comparison to other training components, players do complete similar relative distance during drill-based activities compared to match-play, while also completing greater high-speed running and sprint distance in running drills. Small-sided games and training matches seem the most suitable methods of preparing players for competitive match-play currently, however other methods can and should be utilised, depending on the performance metric coaches wish to develop.

## Figures and Tables

**Table 1:** Definition of each training component.

Warm-up	Any activity or movement with the main aim of preparing the body mentally, physically, and psychologically for the forthcoming session.
Drills	Defined as repetitive tasks, with the focus on improving technical and tactical skills in the absence of opposition.
Small-sided games	Both possession and bi-directional games, with the aim of outperforming an opposing team, with varying pitch sizes, playing numbers, and rules compared to the traditional games.
Running	Any activity with the main focus on improving speed or aerobic endurance through traditional methods with the absence of technical skills.
Training matches	Traditional 15 v 15 games, played on a full-size pitch with standard rules applied.
Transition	Any element of training without the ball in play, such as drink breaks, and explanations or discussion of previous or forthcoming activities.

**Table 2:** The median (interquartile) performance running metrics of elite camogie match-play and training.

	Total Distance (km)	Relative Distance (m∙min^−1^)	Peak Speed (m∙s^−1^)	High-Speed running (m)	Sprint Distance (m)	Acceleration Count (3–4 m∙s^−2^)	Acceleration Count (>4 m∙s^−2^)	Deceleration Count (3–4 m∙s^−2^)	Deceleration Count (>4 m∙s^−2^)
Total match	6.13 (5.57–6.75) *	92 (83–103)	7.22 (6.94–7.62) *	546 (451–674) *	256 (200–331) *	30 (22–38)	8 (5–12)	32 (24–41) *	19 (14–25) *
Total training (ball-in-play)	4.25 (2.75–5.50)	96 (88–104) **	6.66 (6.34–7.00)	481 (286–684)	182 (94–321)	33 (24–42) **	11 (7–17) **	28 (21–36)	15 (9–21)
Training as percent of match data (%)	69	104	92	88	71	110	137.5	87.5	79

* Significantly greater than total training (ball-in-play) (*p* < 0.05). ** Significantly greater than match-play (*p* < 0.05).

**Table 3:** The median (interquartile range) training component relative distances compared in percentage terms to match-play.

	Relative Distance (m∙min^−1^)	High-Speed Running (m∙min^−1^)	Sprint Distance (m∙min^−1^)	Acceleration Count (per min) (3–4 m∙s^−2^)	Acceleration Count (per min) (>4 m∙s^−2^)	Deceleration Count (per min) (3–4 m∙s^−2^)	Deceleration Count (per min) (>4 m∙s^−2^)
Match-play	92 (83–103)	8.29 (6.89–10.27)	3.92 (2.95–5.04)	0.46 (0.34–0.57)	0.12 (0.08–0.18)	0.49 (0.36–0.62)	0.29 (0.22–0.37)
Warm-up	76 (69–84) *	2.75 (1.25–5.17) *	0 (0–0.43) *	0.60 (0.32–0.95) *	0.20 (0–0.36)	0.51 (0.28–0.81)	0.16 (0–0.33) *
Percent of Match Data (%)	83	33	0	129	164	104	55
Drills	96 (76–114)	7.53 (0.80–17.14)	0 (0–3.54) *	1.09 (0.47–2.00) *	0 (0–0.71)	0.67 (0–1.43) *	0 (0–0.57) *
Percent of Match Data (%)	105	91	0	236	0	136	0
Small-sided games	116 (96–134) *	11.29 (5.54–17.23)	1.22 (0–5.33) *	0.75 (0.31–1.33) *	0 (0–0.42)	0.75 (0.31–1.33) *	0.34 (0–0.75)
Percent of Match Data (%)	127	136	31	162	0	154	117
Running	112 (80–140) *	24.36 (10.80–48.44) *	14.53 (4.68–38.64) *	0.82 (0.44–1.29) *	0.50 (0.21–1.07) *	0.27 (0–0.81)	0 (0–0.44) *
Percent of Match Data (%)	122	294	371	177	409	56	0
Training matches	112 (99–122) *	12.17 (9.16–16.18) *	5.17 (2.96–7.86)	0.50 (0.36–0.73)	0.13 (0.06–0.21)	0.55 (0.41–0.77)	0.35 (0.20–0.48)
Percent of Match Data (%)	122	147	132	108	104	112	120

* Significantly different (*p* < 0.05) from match-play.

## Data Availability

The data presented in this study are available on request from the corresponding author.
